# Pyorrhœa Alveolitis in the Elephant

**Published:** 1888-09

**Authors:** 


### Pyorrhœa Alveolitis in the Elephant.

Mr. Edwards has confided to me for examination, a molar of an Asiatic elephant, fallen spontaneously. This tooth was intact, at least in appearance, a fraction of the root having been taken off for examination. It weighed in its dry state 1 kil. .972. The root was covered with a crust of calcareous aspect, of variable thickness but exceeding at certain points 3 or 4 millimeters. The inferior extremity of the root seemed to have been the seat of an intense pathological process and presented sharp prominences, incompatible with the normal state. We have verified by direct examination, as well as by microscopical, that the calcareous crust covering the root, was constituted by the salivary tartar, that is to say, by the micro-organisms having provoked the deposit of calcareous salts held in solution in the saliva. The examination of the lesions has been made comparatively with those of a tooth root of a large molar of an elephant, reputed healthy. The cementum presented all the degrees of alteration, from the most superficial, to their entire disappearance: not only were there micro-organisms on the surface, but they had penetrated into the whole extent of its thickness. In the points where the dentine had been exposed, there were found in the creases, more or less deep, a covering of micro-organisms, more or less compact, with irregular outlines. These micro-organisms had penetrated the canalliculi, and it is possible had penetrated into the depths of the dentine. The lesions did not differ in any degree from those observed in man, and present, with them a striking similarity.

We conclude then the Asiatic elephant in captivity, may be attacked with the same malady which has been described in man, under the name of Gingivitis Infectious. (*Pyorrhœa Alveolitus.*)

